# Dynamic eye avoidance patterns in the high autistic traits group: An eye-tracking study

**DOI:** 10.3389/fpsyt.2023.1086282

**Published:** 2023-03-24

**Authors:** Huiqin Xue, Ludan Zhang, Junling Wang, Wei Liu, Shuang Liu, Dong Ming

**Affiliations:** ^1^Academy of Medical Engineering and Translational Medicine, Tianjin University, Tianjin, China; ^2^Children’s Hospital, Tianjin University, Tianjin, China

**Keywords:** autistic trait, face scanning, dynamic strategy, time course, social attention

## Abstract

**Introduction:**

Reduced fixation to the eye area is the main characteristic of social deficits associated with Autism Spectrum Disorder; a similar pattern may exist in individuals with high autistic traits. However, their scanning patterns to the eye area of emotional faces are still unclear on the time scale.

**Methods:**

In the present study, we recruited 46 participants and divided them into the high autistic traits (HAT) group (23 participants) and the low autistic traits (LAT) group (20 participants) based on their Autism Spectrum Quotient (AQ) scores. Moreover, we captured their eye movement patterns when observing different angular emotional faces. We extracted the proportional fixation time to the eye area under different time windows.

**Results:**

The results showed that the fixation time of the HAT group was always significantly smaller than that of the LAT group (*p* < 0.05), and the difference between the two groups increased in the middle and late stages of face presentation. The results of the linear regression analysis showed that the proportional fixation time was negatively correlated with AQ scores (*p* < 0.05), indicating that the proportional fixation time to the eye area could be a potential indicator to measure the level of autistic traits. We then calculated the latency to orient the eye area and the latency to disengage the eye area to explore the priority of observation of the eyes. The results showed that compared with the LAT group, the HAT group has a longer latency to orient the eye area (*p* < 0.05) and has longer latency to disengage the eye area (*p* < 0.05), illustrating that the HAT group saw the eyes more slowly and left them faster.

## 1. Introduction

Autism Spectrum Disorder (ASD) is a continuum of neural developmental disorders (1). It means that autism-like symptoms can be found in clinical patients and ordinary people, such as the Broader autistic phenotype. ASD represents the upper extreme of severity in the continuous spectrum of symptoms (2–4). Providing mental health support for these people with autism-like symptoms is just as important as providing interventions for children with autism. Thus, researchers have extended the study of autism to a broader range of ordinary people, and the concept of autism characteristics has emerged. Autistic traits are a collection of behavioral characteristics, personality, and cognitive characteristics associated with autism displayed by ordinary people. But the severity of the symptoms of autistic traits is weak are not severe enough for a clinical diagnosis (5, 6). Individuals with high levels of autistic traits are somewhat consistent with ASD patients regarding external behavioral manifestations and internal brain function activities. Social impairment remains at the core manifestation of their impairment (7).

Atypical social attention is one of the earliest and most important manifestations of social impairment in people with high autistic traits. Social information can strongly capture and guide the attention of typically developing individuals, especially face information. Faces are complex biological and sociological stimuli that play a fundamental supporting role in social interactions (8–10). Studies have shown that typically developing people are particularly attracted to faces in infancy (11). However, individuals with high autistic traits have some atypicality in their attention to social information, mainly reflected in a weakened preference for social information, e.g., face stimuli (12–14). Many findings have suggested that individuals with high autistic traits did not have a preferential orientation and attention preference for faces. The condition is also present in happy and threatening face stimuli (15–18). Some results from eye-tracking technology demonstrated that individuals with high autistic traits scan faces differently from individuals with low autistic traits, when they observed the dynamic videos, their visual search for the face was reduced (19). Vabalas et al. (20) found that during face-to-face interaction, individuals with high autistic traits exhibited reduced visual exploration of the faces. Moreover, it is noteworthy that they also found that visual exploration was related to autistic traits; the higher the level of autistic traits, the lower the glance frequency to the face.

The atypical scanning pattern on the eyes is the main feature of face scanning in individuals with high autistic traits. The eyes are the more social part of the face transmitting a wealth of information about the mental state of others (21) and playing an irreplaceable role in social adaptation and interpersonal cognitive processes. Several eye-tracking studies have examined the fixation on the eye area in individuals with high autistic traits. Siblings with autism often have higher levels of autistic traits. Dalton et al. (22) found that during the face-processing task, the siblings with autism showed decreased gaze fixation to the eyes compared with the control group, which was similar to that of the autism. The atypical eye scanning was also shown in individuals with high autistic traits in non-autistic relatives. When watching the directed and averted video, individuals with low autistic traits prefer the directed eyes; however, individuals with high autistic traits do not (23).

The eye avoidance of individuals with high autistic traits may be one of the reasons for their social impairment. Peter et al. (24) believed that when individuals do not pay enough attention to social information and are unable to take in and process it properly, it is difficult for individuals to accumulate enough social learning experiences, leading to insufficient social development and eventually leading to social impairment. For example, they show impairment in recognition of facial identity and facial emotion (25–27). However, the selection of appropriate interventions requires an understanding of the mechanisms of eye avoidance in groups with high autistic traits. There are two theories that explain the mechanism behind eye avoidance. The “gaze aversion hypothesis” (28) is that stimuli with more information, such as faces and eyes, can lead to high physiological arousal, and active avoidance of such information is used to alleviate the discomfort caused by this high arousal (18, 29, 30). The “gaze indifference hypothesis” (31) means that individuals are insensitive to social information and consider the information in it to be useless (32, 33). Both hypotheses have been widely validated. These two hypotheses can be tested to some extent using the individual’s gaze priority for the eyes (34). The “gaze aversion hypothesis” is supported if subjects shift their gaze more quickly after seeing the eyes, and the “gaze indifference hypothesis” is supported if subjects look more slowly at the eyes after the face is presented.

Due to the importance of social attention, intervention training for individuals with high autistic traits is often conducted by using guidance to focus more on social information, e.g., eyes. Based on the strong association between reduced fixation to the eyes and the core performance in individuals with high autistic traits, understanding the processing of the group is critical to our understanding of their social impairment at its source. At the same time, this has important implications for the selection of appropriate interventions for autism and the high autistic traits people at the sub-clinical level.

However, most existing research explored the fixation time to the eyes based on the total amount of time, which may lose information on the change in individuals’ gaze points over time. The scanning patterns of individuals with high autistic traits to the eye area of emotional faces are still unclear on the time scale. Therefore, one of the main aims of the present study was to investigate when the pattern of eye avoidance emerges and how the pattern changes over time in individuals with high autistic traits. Furthermore, in daily life, face stimuli are not just presented in one expression or one state, so we wanted to observe the gaze to the eye area in a condition with better ecological validity for individuals with high autistic traits. To achieve these aims, we designed a free exploration experiment with multi-angle emotional faces. When the participants observed the face stimuli, we recorded and analyzed the subjects’ eye movement signals to reveal the dynamic scanning strategies of people with high autistic traits.

## 2. Method

### 2.1. Participants and ethical considerations

The present study used G*power 3.1 (35) to calculate the total sample size. The effect size *f* was set to 0.25, and α was set to 0.5; the results showed that a total of 14 subjects were required to achieve 95% statistical test power. In the present study, 43 college students, including 25 boys (*M* = 23.34, SD = 2.47) and 18 girls (*M* = 23.89, SD = 2.16), were recruited to participate in the experiment. All subjects were right-handed, with normal visual acuity or corrected visual acuity, without color blindness or color weakness, and excluded from serious intracranial injury, drug abuse, psychiatric history, or family history. This study ensured that all subjects participated actively without knowing the experimental objectives and signed informed consent. Ethical approval was obtained from the Children’s Hospital of Tianjin University Ethics Committee.

The Autism Spectrum Quotient (AQ) (36) is a powerful tool for assessing the distribution of autistic traits level among the general population and of favorable reliability and validity. It is organized into five subscales and 50 items in total. Since compared the binary scoring method (1-1-0-0), the 4-point Likert’s scoring (4-3-2-1) could obtain a better approximation of continuous distribution (37) to capture individual variability better (38), we used the latter to retain more information about the answers (1), expand the score range, and distinguish the difference between individuals. In addition, we used the total score as the analysis object. Individuals with relatively high scores on the AQ scale have social cognitive deficits similar to those of patients to a certain extent. In the present study, Cronbach’s α of the total AQ and the subscales are 0.794, 0.839 (Social Skill), 0.614 (Attention Switching), 0.667 (Attention to Detail), 0.766 (Communication), 0.465 (Imagination). According to the comparative studies (14, 18), we used the average of the total scores of the initial group (43 subjects) as the critical score (*M* = 114.163) to divide all subjects into the high autistic traits (HAT) group and the low autistic traits (LAT) group. On this basis, we examined the score differences (*t*-test) between the HAT and the LAT groups under the AQ scale and the other subscales. In addition, we also performed statistical tests for age (*t*-test) and gender (chi-square test) for the participants of the two groups. The participant characteristics are presented in Table 1.

**Table 1:** Participant characteristics.

	HAT group (*n* = 23)	LAT group (*n* = 20)	*t*/*χ*^2^-value	*p*-value
Age (years): *M* (SD)	23.84 (2.37)	23.26 (2.30)	0.816	0.419
Gender: male/female	13/10	12/8	0.053	0.818
*AQ, total score: M* (SD)	124.435 (7.191)	102.350 (6.385)	−10.577	< 0.001
*AQ*, Social Skill*: M* (SD)	25.609 (2.935)	17.250 (3.905)	−7.997	< 0.001
*AQ*, Attention Switching*: M* (SD)	27.565 (3.396)	23.250 (3.291)	−4.216	< 0.001
*AQ,* Attention to Detail*: M* (SD)	25.739 (3.545)	25.700 (5.121)	−0.029	0.977
*AQ,* Communication*: M* (SD)	23.000 (4.200)	17.500 (3.561)	−4.593	< 0.001
*AQ,* Imagination*: M* (SD)	22.522 (3.475)	18.650 (2.889)	−3.937	< 0.001

The results of the independent-sample *t*-test showed that there was a significant difference in the total scores and four subscales scores (except the Attention to Detail subscale) of the HAT group and the LAT group (*p* < 0.001). The results show that the experimental grouping is effective, and the subjects in the HAT group are representative.

### 2.2. Materials and apparatus

#### 2.2.1. Stimuli materials

The visual stimuli used in the present study from the Karolinska Directed Emotional Faces (Lundqvist, 1998) stimulus set. Existing researchers mostly focused on single facial expressions (e.g., neutral) and faces from a single angle (frontal faces) and neglected the possible influence of expressions and face angles. Whether the eye avoidance of individuals with high autistic traits is also present in other angles and emotions of the face remains to be explored. Thus, we selected facial stimuli from 10 women and 10 men, each displaying four different emotional expressions (happy, neutral, sad, and angry), each expression being photographed from five different directions (three angles: 0°, 45°, 90°). In the present experiment, we used 400 face pictures as a visual stimulus.

#### 2.2.2. Eye-tracking system

A Tobii Pro Spectrum 1,200 binocular eye tracker and Tobii Prolab software were used to record gaze position and pupil diameter, which were measured at 1200 Hz (one sample every 0.83 ms). The Tobii Pro spectrum 1,200 eye tracker allows subjects to move their heads within a certain range. Facial stimuli sequences of 562 × 762 pixels resolution were presented using the Psychtoolbox of MATLAB on a 27-inch screen (1,920 × 1,080 pixels resolution, refresh rate of 120 Hz).

### 2.3. Experimental design and testing procedure

All participants sat approximately 60 cm from the screen and received a practice block before the experiment to gain familiarity with the task. Then, we used Tobii’s 5-point calibration method for every subject. We accepted the calibration only if the error vectors were smaller than 0.5 degrees of visual angle; otherwise, we needed to calibrate again until the calibration was successful.

**Figure 1:**
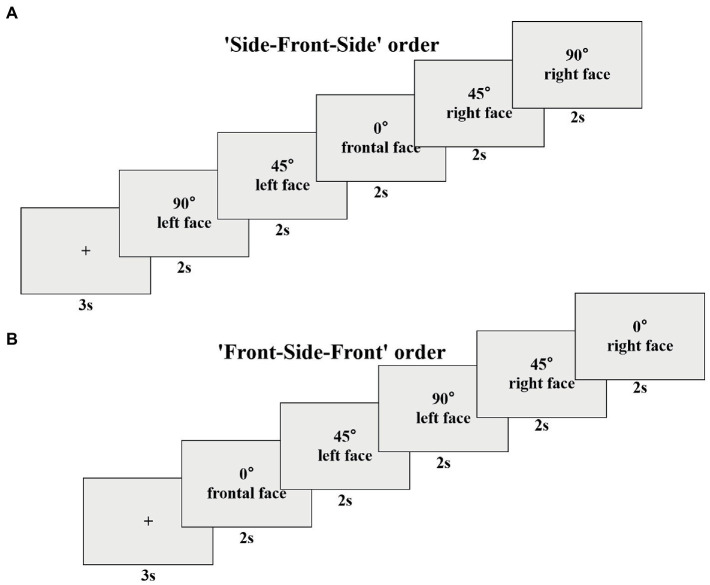
The possible trial of the ‘Side-Front-Side’ order **(A)** and the ‘Front-Side-Front’ order **(B)**.

After successful calibration, the experiment officially began. First, a cartoon picture appeared in the center of the screen for 3 s as a gazing point to attract the subjects’ attention. Subsequently, five pictures of faces appeared in succession, which were pictures of the same expression from different angles. Each picture was presented for 2 s, and the subjects were instructed to observe it freely. In order to simulate faces in real social situations, we designed a free scanning experiment of multi-angle emotional faces with more ecological validity. According to the gradual change of the presentation angle, there were two presentation sequences of the five face pictures in the experiment, one was ‘Side-Front-Side’, and the other was ‘Front-Side-Front’. The schematic representation of the ‘Side-Front-Side’ order was ‘90° → 45° → 0° → 45° → 90°’ (Figure 1A), the schematic representation of the ‘Front-Side-Front’ order was ‘0° → 45° → 90° → 45° → 0°’ (Figure 1B). By setting such two presentation sequences, the present study could balance the influence of the orders. There were five blocks in the whole experiment, and each block contained 16 trials. The playback time of a block was approximately 3 min, and the complete playback of all blocks was approximately 15 min. In the process of stimulus presentation, the two presentation orders, the people’s faces, and the facial expressions were randomized.

### 2.4. Data analysis

#### 2.4.1. Pre-processing

In this study, Tobii Pro lab software was used to capture the oculomotor data by recording the screen. At the end of the experiment, raw data with gaze point coordinates, time stamps, event values, and pupil diameters were exported from the software. The filter we used was the Tobii I-VT (Attention) (39) filter with a default threshold of 100 degrees per second (100°/s). Then, to prevent longer fixation points from being split into shorter fixation points due to data loss or noise, we need to merge adjacent fixations. Due to missing data larger than 75 ms generally representing the subjects’ blink (40), according to previous studies requiring merged fixations (40–44), spatially and temporally adjacent gaze points were combined (<0.5°, <75 ms). Finally, we referred to previous studies (40, 43, 45) and discarded short fixations (less than 60 ms) that were not meaningful since the eyes and brain require some time for the information to be processed (46). After pre-processing the data, we changed the labels of the raw data in MATLAB, and the gaze data under different conditions were intercepted for subsequent analysis to facilitate our subsequent analysis in MATLAB and Python.

#### 2.4.2. Time course analysis

Our main purpose in the present study is to explore how the fixation duration on the eyes varied over a shorter time window in the HAT vs. LAT groups when observing faces. First, we calculated the mean proportion of fixation time (fixation time to the eye area (s) / fixation time to the face area (s)) in the eye area in each presentation order (5 × 2 s). Based on this, we divided data obtained during the single-face image (2 s) into 5-time windows. In every 0.4-s time window, we calculated the mean proportion of fixation time to create time series signals in the eye area.

On this basis, this study conducted a repeated measures analysis of variance (ANOVA) with the proportional fixation time as the dependent variable, group (HAT, LAT) as a between-participants factor, and the emotion (happy, neutral, sad, angry), time course (TC1, TC2, TC3, TC4, TC5) as within-participant factors, to reveal how fixation time to the eyes is changed in different time course in the HAT and LAT groups.

#### 2.4.3. Priority of observation in the eye area

To quantify the degree of preference for the eye area in the HAT and LAT groups, we calculated the latency to orient the eye area and the latency to disengage the eye area.

The latency to orient the eye area was defined as the time difference between the presentation of the emotional face and the first time the subject’s gaze point fell into the eye area. If the subject did not look at the eye area during the picture presentation, the index was defined as 2 s (total time of the picture presentation).

The latency to disengage from the eye area was defined as the duration of the first time the subject’s gaze point fell into the eye area. The index was defined as 0 s if the subject did not look at the eye area. The shorter the time to disengage from the eye area, the more the individual showed eye avoidance.

On this basis, this study conducted a repeated measures ANOVA with the latency to orient the eye area and the latency to disengage from the eye area, respectively, as the dependent variable, group (HAT and LAT) as a between-participants factor, and the emotion (happy, neutral, sad, angry), angle (90°, 45°, 0°) as within-participant factors, to reveal how fixation time to the eyes are changed in different time course in the HAT and LAT groups.

## 3. Results

### 3.1. Dynamic time-course changes

People observe faces dynamically, therefore, we calculated the changes in proportional gaze time in the eye area of the HAT and LAT groups under the ‘Side-Front-Side’ and ‘Front-Side-Front’ orders.

**Figure 2:**
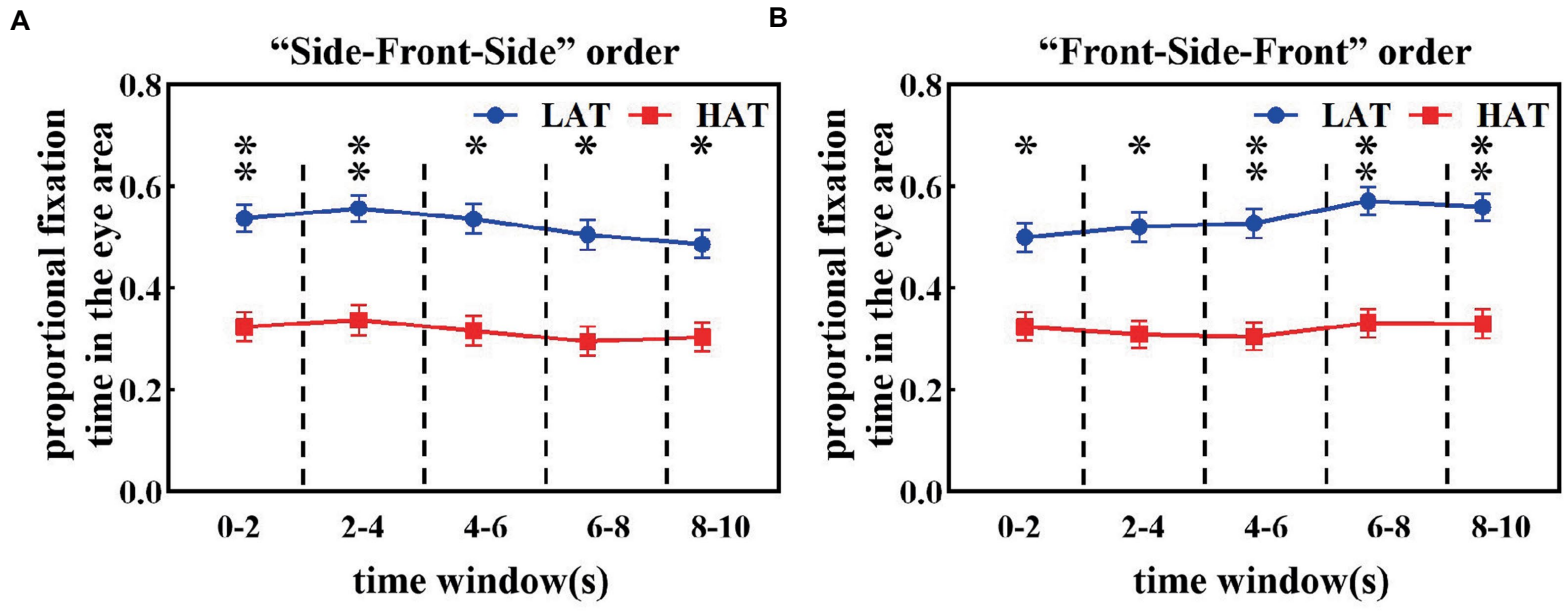
The proportional fixation time in the eye area under the ‘Side-Front-Side’ order **(A)** and ‘Front-Side-Front’ order **(B)** of the HAT and the LAT groups.

Figures 2A,B shows the proportional fixation time in the eye area during each 2 s time window of HAT and LAT groups in the ‘Side-Front-Side’ and ‘Front-Side-Front’ orders. As can be seen from the figures, the proportion of fixation time in the eye area was smaller in the HAT group than in the LAT group for both presentation orders. The repeated measures ANOVA revealed a significant main effect of the group under the ‘Side-Front-Side’ order (*F*(1,41) = 7.092, *p* = 0.011, *η_p_^2^* = 0.147) and the ‘Front-Side-front’ order (*F*(1,41) = 7.942, *p* = 0.007, *η_p_^2^* = 0.162).

These results indicated that the difference between the HAT and the LAT groups was significant. In observing the different angular emotional faces, the HAT group always showed eye avoidance.

**Figure 3:**
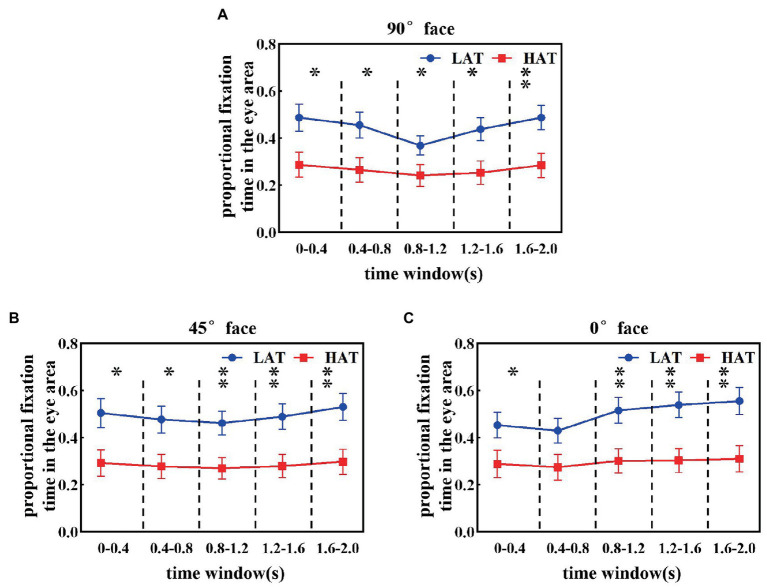
The proportional fixation time of eye areas in the 90° **(A)**, 45° **(B)**, and 0° **(C)** face of the HAT and the LAT groups within *5 time* courses.

Furthermore, to reveal how eye avoidance in the HAT group changes over time, we calculated the proportional fixation time of the HAT and LAT groups in the eye area in a smaller time window (every time window is 0.4 s). The results are represented in Figure 3. From the resultant picture, we can find that in all time courses, the proportional fixation time of the HAT group was smaller than that of the LAT group. The repeated measures ANOVA revealed that angle, time course, and the group have significant three-way interaction (*F*(8,328) = 3.357, *p* = 0.001, *η_p_^2^* = 0.076), indicating that the HAT group spent significantly less time looking at the eyes than the LAT group throughout the presentation of the face pictures. In addition, we also found that the change trends of the two groups were different.

As shown in Figure 3A, when observing the 90° face, the proportional fixation time of the LAT group in the eye area showed a trend of first decreasing and then increasing. Specifically, the proportional fixation time of the eye area for the LAT group did not show significant differences in time courses 1 and 2, decreased significantly in time course 3 (*p* < 0.05), and increased significantly in time courses 4 and 5 (*p* < 0.05). For the HAT group, the proportional fixation time of eye area was essentially the same for the first four time courses, appearing elevated at time course 5 and significantly greater than time courses 3 and 4 (*p* < 0.05).

The proportional fixation trends of the HAT and the LAT groups in the 45° face within 5 time courses are represented in Figure 3B. In the first four time courses, the proportional fixation time of the LAT group was the same and increased in time course 5 and was significantly greater than in time courses 3 and 4 (*p* < 0.05). For the HAT group, the proportional fixation time was stable within 5 time courses.

From Figure 3C, we can see that when observing the 0° face, the proportional fixation time in the LAT group increased significantly at time course 3 (*p* < 0.05) and continued until the end of the face presentation. For the HAT group, the proportional fixation time remained stable within 5 time courses.

The previous analyses demonstrated that the HAT group consistently avoided the eyes and the difference between the two groups increased in the middle and late stages of face presentation (starting from the time course 3).

### 3.2. The potential indicator measuring the degree of autistic traits

**Figure 4:**
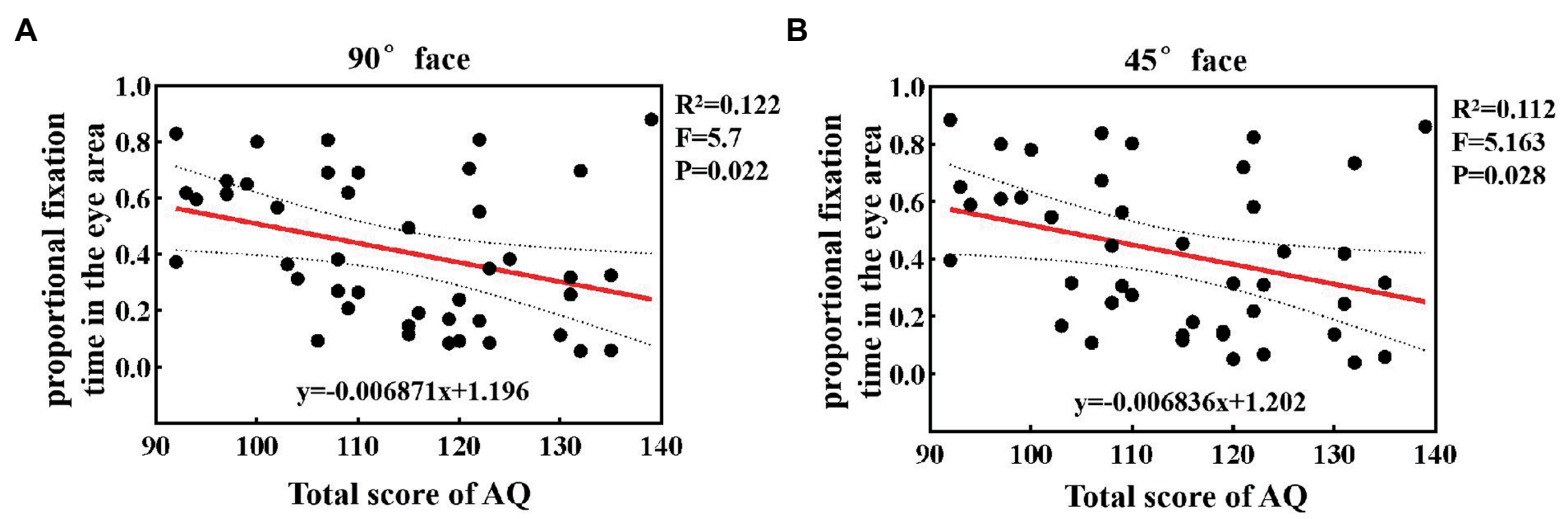
The linear regression analysis between autistic traits and the proportional fixation time in the eye area at 90° **(A)** and 45° **(B)** faces.

To quantify specific relations between autistic traits and the proportional fixation time, we calculated the correlation coefficient and performed a linear regression analysis between them under different angular faces. The results at 90° and 45° faces are shown in Figure 4. It was found that when observing 90° and 45° faces, as the individual’s total AQ score increased, the proportional fixation time in the eye area showed a decreasing trend. Furthermore, the statistical results showed a significant negative correlation between the two factors (*p* < 0.05). Under 0° and 45° faces, 12.2 and 11.2% of the phenomenon of avoiding eyes can be explained by the level of autistic traits, with *R^2^* being 0.122 and 0.112, respectively. For 0° face, there was also a negative correlation between the proportional fixation time and the total score and reached the marginal significance (*p* = 0.056).

The aforementioned results demonstrated that the duration of gaze on the eye area was related to the level of autistic traits. The higher the level of autistic traits, the shorter the fixation time of the eye area, and the more likely it is to show eye avoidance.

### 3.3. The latency to orient the eye area

**Figure 5:**
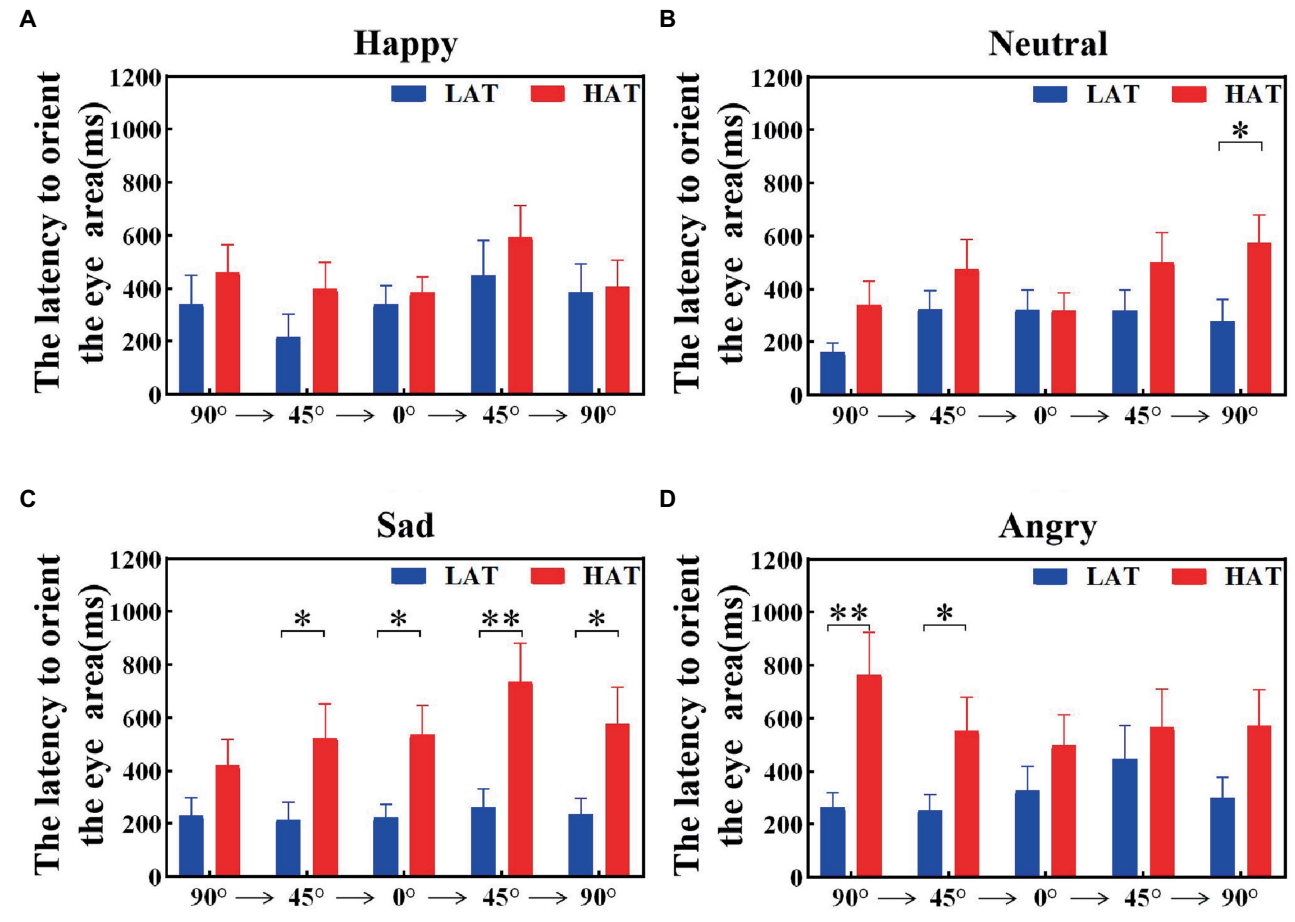
The latency to orient the eye area of the HAT and the LAT groups during the observation of happy **(A)**, neutral **(B)**, sad **(C)**, and angry **(D)** emotional faces with different angles under the ‘Side-Front-Side’ order.

**Figure 6:**
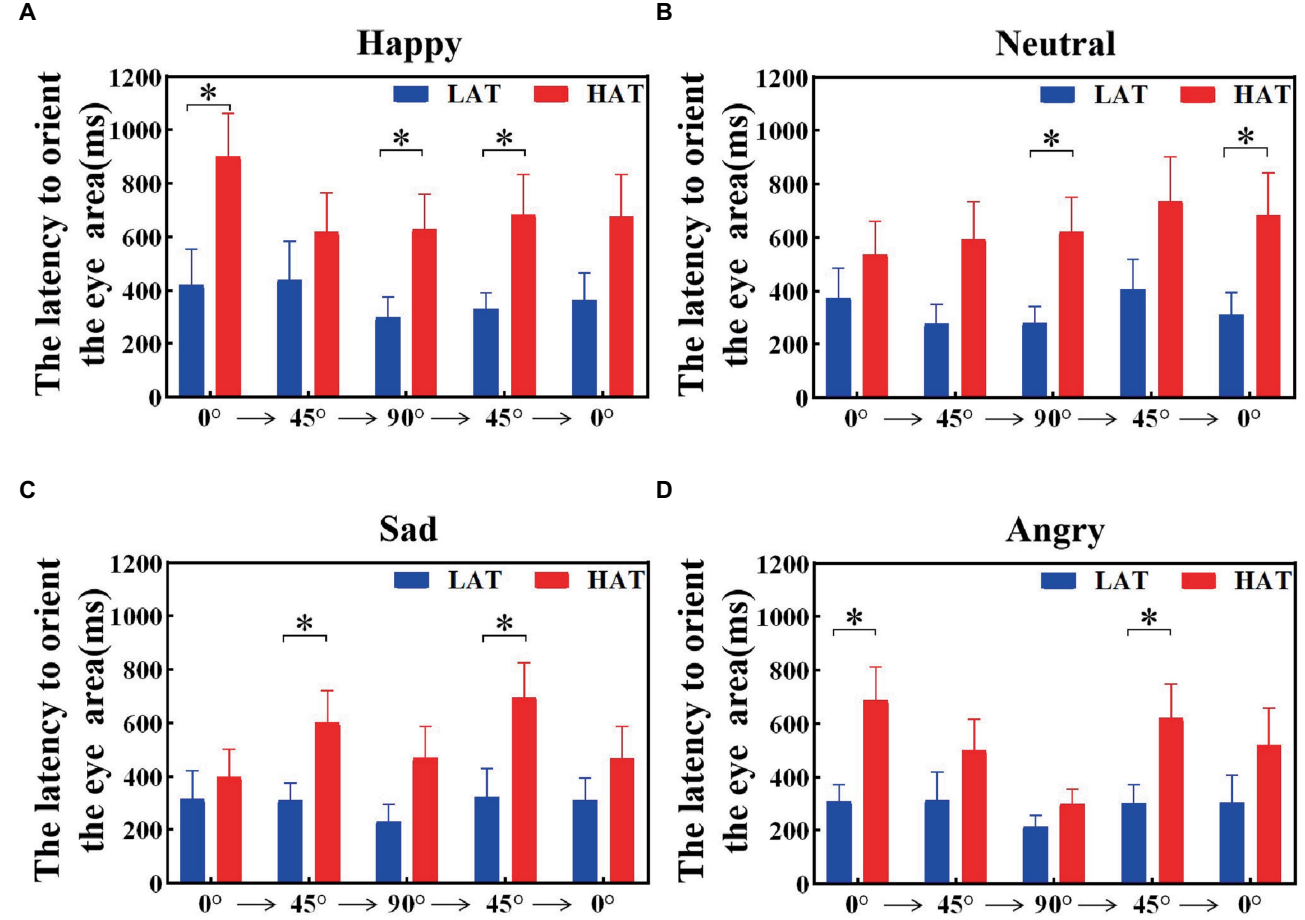
The latency to orient the eye area of the HAT and the LAT groups during the observation of happy **(A)**, neutral **(B)**, sad **(C)**, and angry **(D)** emotional faces with different angles under the ‘Front-Side-Front’ order.

The latency to orient the eyes was related to the sensitivity of the eyes. To explore the velocity of locating eyes in the HAT and LAT groups, we calculated the latency to orient the eye area of the HAT and LAT groups under two presentation orders separately. The results are represented in Figures 5, 6. According to the information in the figures, we can see that the latency to orient the eye area of the HAT group is greater than that of the LAT group. The result of repeated measures ANOVA showed that the main effects of the group were significant in the ‘Side-Front-Side’ order (*F*(1,41) = 93.175, *p* = 0.013, *η_p_^2^* = 0.140) and the ‘Front-Side-Front’ order (*F*(1,41) = 80.579, *p* = 0.010, *η_p_^2^* = 0.150). It revealed that the latency to orient the eye area was significantly longer in the HAT group than in the LAT group. That is, compared to the LAT group, the HAT group would look at the eyes more slowly, and there was atypical eye orientation.

The aforementioned analysis showed that compared to the LAT group, the HAT group had a longer latency to orient the eye area and oriented the eyes more slowly after the emotional face appeared.

### 3.4. The latency to disengage from the eye area

**Figure 7:**
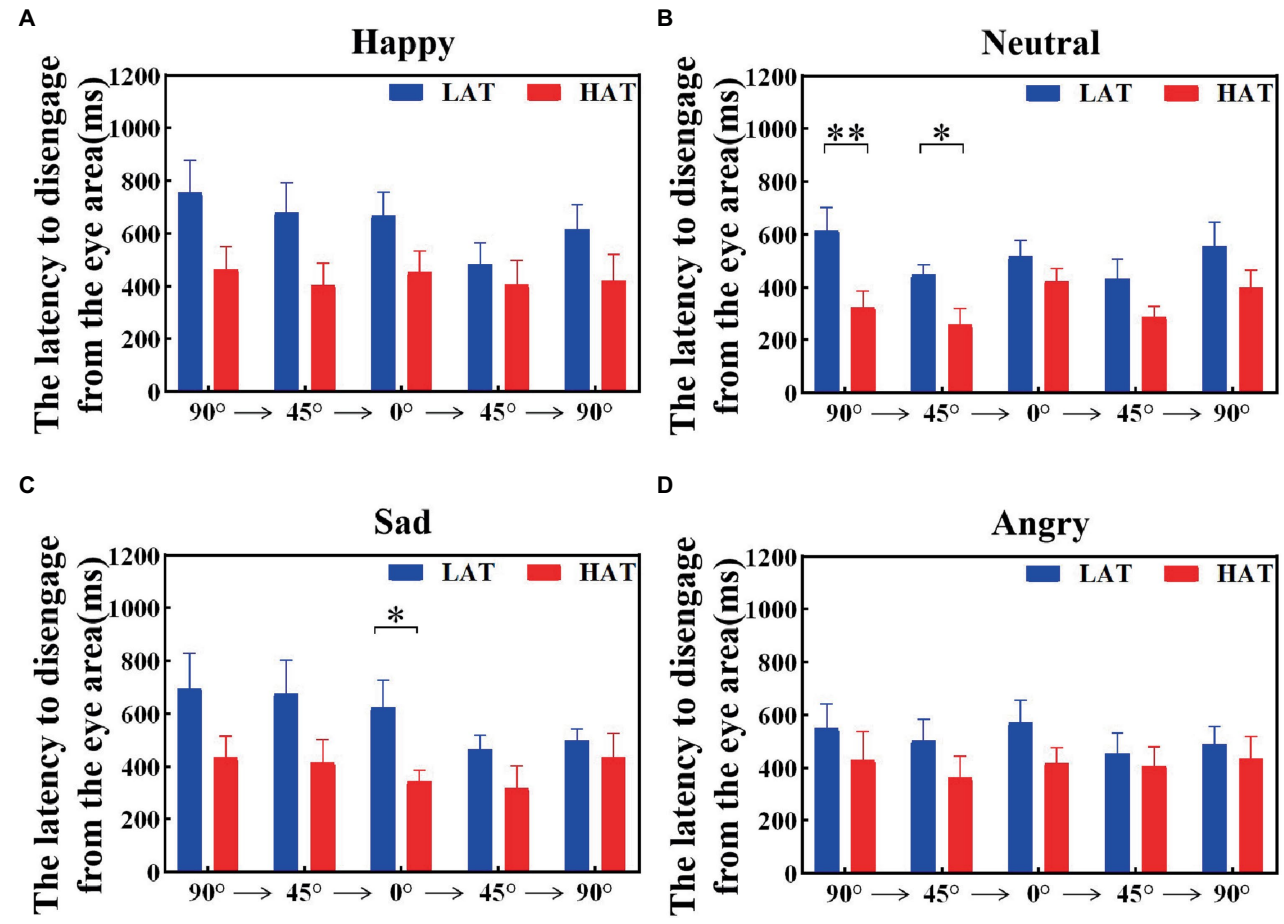
The latency to disengage from the eye area of the HAT and the LAT groups during the observation of happy **(A)**, neutral **(B)**, sad **(C)**, and angry **(D)** emotional faces with different angles in the *‘Side-Front-Side’* order.

**Figure 8:**
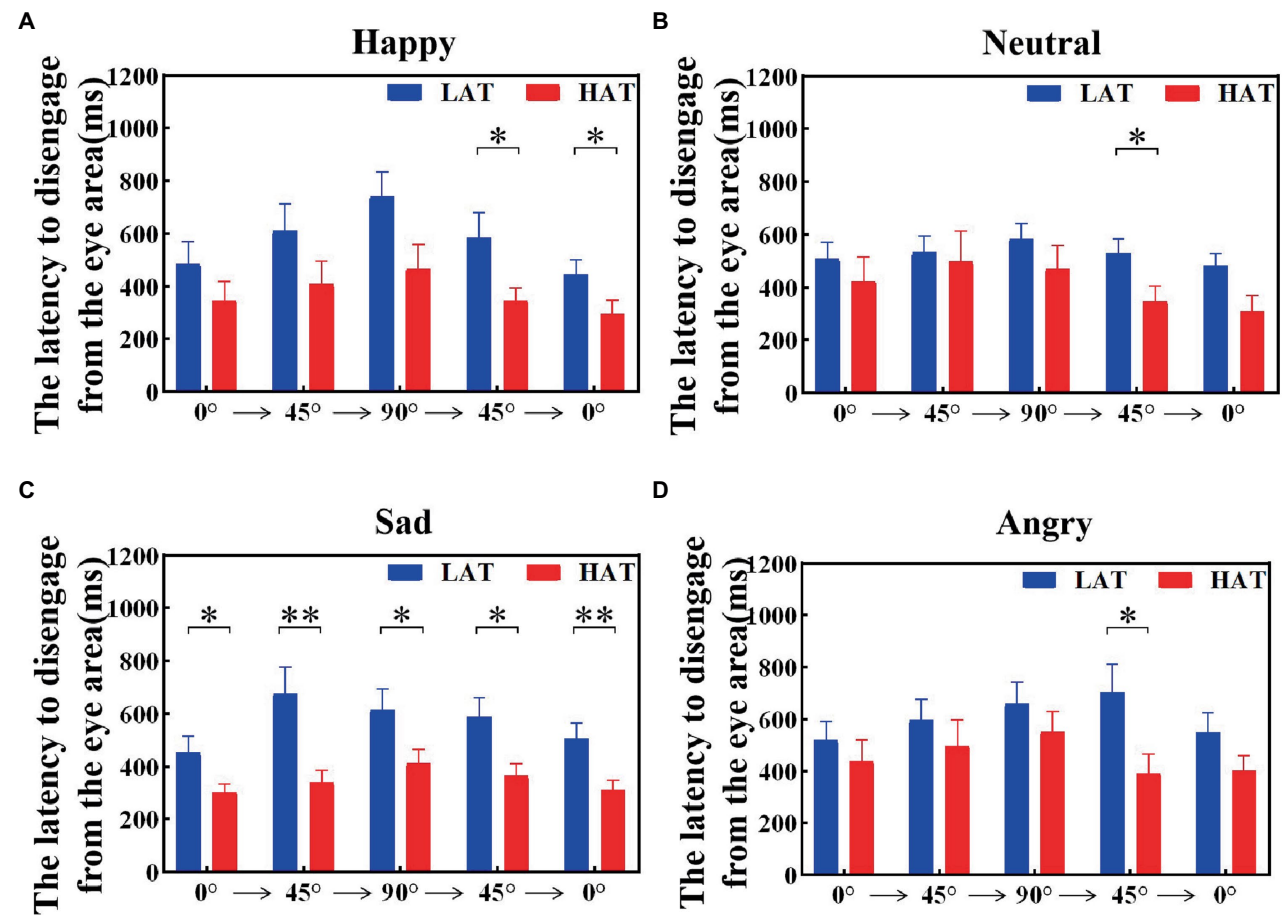
The latency to disengage from the eye area of the HAT and the LAT groups during the observation of happy **(A)**, neutral **(B)**, sad **(C)**, and angry **(D)** emotional faces with different angles in the *‘Front-Side-Front’* order.

The latency to disengage from the eye area was related to the tendency to avoid the eyes. To investigate the visual shift rate between the HAT group and the LAT group after seeing the eye area, we calculated the latency to disengage from the eye area, that is, the duration of the first fixation point in the eye area. The results are represented in Figures 7, 8. The results showed that under both presentation orders, the latency to disengage from the eye area of the HAT group is smaller than that of the LAT group. The repeated measures ANOVA revealed significant main effects of the group both in the ‘Side-Front-Side’ order (*F*(1,41) = 4.803, *p* = 0.034, *η_p_^2^* = 0.105) and the ‘Front-Side-Front’ order (*F*(1,41) = 5.966, *p* = 0.019, *η_p_^2^* = 0.136), indicating that the latency of disengage from the eye area in the HAT group was significantly smaller than that in the LAT group, the HAT group shifts their vision more quickly after looking into the eye area than the LAT group.

The above analyses showed that compared to the LAT group, the HAT group oriented the eye region more slowly when observing different angular emotional faces. Moreover, after looking into the eye area, the HAT group shifted their vision more quickly.

## 4. Discussion

Eye avoidance is a common manifestation of the HAT group in social interaction and a possible source of their social impairment. However, the existing studies did not reveal when eye avoidance in the HAT group emerged and how it changed over time. To solve these problems, we divided the participants into the HAT and LAT groups by the total AQ score and examined the differences in the dynamic scanning strategy in the two groups.

Our analysis was based on valid grouping. In this study, the total AQ score and the four subscales scores of the HAT group are significantly higher than those of the LAT groups. However, there was no significant difference in the scores of the two groups on the Attention to Detail subscale. This result may be due to cultural differences between East and West that affect the assessment of this dimension (47). In China, many people have a preference for using lucky Chinese numbers, e.g., 8 and 6, and they will subconsciously pay attention to the lucky number on the license plate. However, in Western countries, the attention to the strings of information may be the embodiment of individuals with high autistic traits. It was also found in another study that subjects’ scores on the subscale may not relate to autistic traits (48). Overall, based on significant differences between the two groups under the total AQ and other subscales, the results from the scale still suggest that grouping is valid; the HAT group had a higher level of autistic traits.

Individuals with high autistic traits usually did not look each other in the eye in everyday social interaction. Consistent with existing studies (21–23), our results also suggest that the HAT group did show a reduction in the fixation on the eye area. ASD children have also been found in many clinical studies to exhibit eye avoidance when observing faces (49). This may be common to individuals with high autistic traits and varies with the level of the autistic traits. We found that there was a significant negative correlation between the total AQ score and the proportional fixation time on the eye area at different angles of face stimuli. Thus, we infer that the proportional fixation time of the eye area could predict the level of autistic traits. The smaller the fixation time, the higher the level of autistic traits. This also means that autistic traits extend into the ordinary population and not all or nothing (Constantino and Todd, 2003b, (4)). Furthermore, the present study also has some clinical value. In the context of face recognition, the fixation time of the eye area may be used as a potential marker for measuring the degree of autistic traits. When observing the face, the lower the fixation preference of the eye area, the higher the degree of autistic traits. We hypothesize that the atypical face-scanning strategy could also be used as a potential marker for identifying ASD children (49, 50). These fixation time results also suggest that for the HAT group, we can intervene by increasing their fixation time on the eyes. At the same time, that would allow psychologists and researchers to take the fixation time of the eyes as a measurement to evaluate the effect of intervention therapy. However, we found that although there was a significant correlation, the variance was only approximately 0.12 in the linear regression analysis, which may be due to the large individual variability of the subjects.

Understanding why individuals with high autistic traits avoid the eyes is an important proposition. The “gaze aversion hypothesis” suggests possible reasons for eye avoidance in terms of the “high arousal” of individuals with high autistic traits. From the result of the fixation time trend, we found that the HAT group’s eye-avoidance behavior was present not only for some time but also consistently throughout the face observation. Moreover, the difference between the two groups increased from 1,200 ms after the picture appeared. It shows that the eye avoidance of the HAT group does not weaken with time but shows a stronger pattern. To some extent, this result supports the “gaze aversion hypothesis.” Another significance of exploring dynamic scanning strategies for the eyes in individuals with high autistic traits is that it can help us to distinguish autistic traits (including autism) from individuals with other mental illnesses. For example, social phobia often avoids the eyes (51, 52). Furthermore, the latency to disengage from the eye area supports the hypothesis. We found that the HAT group disengage from the eye area faster than the HAT group. The ‘gaze indifference hypothesis’ is mainly based on the ‘low arousal’ of individuals with high autistic traits. Similar to the study by Kleberg et al. (53), in the present study, we found that the HAT group took a long time to orient toward the eyes, which supports the ‘gaze indifference hypothesis’. In summary, our results support both the ‘gaze aversion hypothesis’ and ‘gaze indifference hypothesis’. Our study also revealed specific patterns of eye avoidance in the HAT group on the time scale. In the initial time window, the HAT group’s eye avoidance was mainly manifested by a larger latency period with a shorter gaze duration. When observing face stimuli, the HAT group will look at the eyes more slowly but will move away from the eyes more quickly than the LAT group after the fixation visit to the eye area. These phenomena also represent the HAT group’s degree of preference for eyes.

Notably, the small ANOVA effect sizes in our results suggest that only a small portion of the phenomenon of avoidance of the eye can be explained by the high level of autistic traits. The difference between the HAT and LAT groups was small. This could be due to the fact that the HAT group had accumulated some experience in social learning that obscured their characteristics, resulting in greater heterogeneity of subjects within the group. There may also be other factors that influenced the subjects’ gaze. In future studies, attention could be paid to reducing the influence of “noise,” such as the hairstyles in facial stimuli.

In brief, we considered the specific dynamic strategies of eye avoidance in the HAT group from the perspective of the time course and the priority of eye gaze, which complemented the social attention of the HAT group to the eyes. The present study has a certain significance in the clinic and theory. The present study supplemented the specific pattern model of eye avoidance in the HAT group; the methodology and results can be extended to clinical ASDs. The atypical dynamic scanning strategy for eye area may be an important bridge to explore the pathological mechanisms of clinical ASDs and individuals with high autistic traits. At the same time, our research also provides the theoretical basis and technical support for the effective intervention of individuals with high autistic traits and clinical ASDs. For these groups, we can intervene to treat them by trying to enhance their attention to social information, e.g., eyes (54, 55). To some extent, it also reflects the reasons for their eye avoidance.

However, there is still room for improvement in our study. For example, we can extend the subject sample to explore the changes in fixation duration of the HAT group on the eye area in a smaller time window. In addition, our study relied on a pre-defined area of interest, which may lose information about the location of the fixation points within the region. Thus, a new alternative method can be found, or a more refined area of interest can be pre-defined to explore the specific differences between the HAT and LAT groups when observing faces in future studies. In addition, we can expand our exploration of the factors influencing the phenomenon of eye avoidance to understand how these factors affect facial scanning strategies with autistic traits.

## Data availability statement

The raw data supporting the conclusions of this article will be made available by the authors, without undue reservation.

## Ethics statement

The studies involving human participants were reviewed and approved by The Children’s Hospital of Tianjin University Ethics Committee. The patients/participants provided their written informed consent to participate in this study.

## Author contributions

HX: acquisition of data, analysis, and drafting the manuscript. LZ: interpretation of data, experiment design. JW: acquisition of data. WL: revising the manuscript critically for important intellectual content. SL: conception and design of study, revising the manuscript critically for important intellectual content. DM: revising the manuscript critically for important intellectual content. All authors contributed to the article and approved the submitted version.

## Funding

The author(s) declare financial support was received for the research, authorship, and/or publication of this article. This research was funded by Tianjin Key Technology R&D 21JCYBJC00360.

## Conflict of interest

The authors declare that the research was conducted in the absence of any commercial or financial relationships that could be construed as a potential conflict of interest.

## Publisher’s note

All claims expressed in this article are solely those of the authors and do not necessarily represent those of their affiliated organizations, or those of the publisher, the editors and the reviewers. Any product that may be evaluated in this article, or claim that may be made by its manufacturer, is not guaranteed or endorsed by the publisher.
