# Corrigendum: Dynamic eye avoidance patterns in the high autistic traits group: an eye-tracking study

**DOI:** 10.3389/fpsyt.2024.1543460

**Published:** 2024-12-24

**Authors:** Huiqin Xue, Ludan Zhang, Junling Wang, Wei Liu, Shuang Liu, Dong Ming

**Affiliations:** ^1^ Academy of Medical Engineering and Translational Medicine, Tianjin University, Tianjin, China; ^2^ Children’s Hospital, Tianjin University, Tianjin, China

**Keywords:** autistic trait, face scanning, dynamic strategy, time course, social attention

In the published article, there was an error in the **Funding** statement. The funding information was inadvertently omitted from the published article. The correct **Funding** statement appears below.

“The author(s) declare financial support was received for the research, authorship, and/or publication of this article. This research was funded by Tianjin Key Technology R&D 21JCYBJC00360.”

The authors apologize for this error and state that this does not change the scientific conclusions of the article in any way. The original article has been updated.

